# Genetic Diagnoses Among Congenital Anomaly Cases in Europe: Data From the EUROCAT Network

**DOI:** 10.1111/ppe.70099

**Published:** 2025-11-24

**Authors:** Jorieke E. H. Bergman, Annie Perraud, Ester Garne, Ingeborg Barisic, David Tucker, Elisa Ballardini, Lea Bruneau, Clara Cavero‐Carbonell, Ianis Cousin, Miriam Gatt, Katya Kovacheva, Anna Latos‐Bielenska, Mary O'Mahony, Isabelle Monier, Isabelle Perthus, Riccardo Pertile, Anke Rissmann, Florence Rouget, Michelle Santoro, Joanna Sichitiu, Christine Verellen‐Dumoulin, Wladimir Wertelecki, Diana Wellesley, Joan K. Morris

**Affiliations:** ^1^ Eurocat Northern Netherlands, Department of Genetics University of Groningen, University Medical Center Groningen Groningen the Netherlands; ^2^ European Commission, Joint Research Centre (JRC) Geel Belgium; ^3^ Department of Pediatrics and Adolescent Medicine Lillebaelt Hospital, University Hospital of Southern Denmark Kolding Denmark; ^4^ Medical School University of Zagreb Zagreb Croatia; ^5^ Research Data & Digital Directorate Public Health Wales Swansea UK; ^6^ Department of Medical Sciences, Neonatal Intensive Care Unit University Hospital of Ferrara, University of Ferrara, IMER Registry (Emilia Romagna Registry of Birth Defects) Ferrara Italy; ^7^ The Reunion Registry of Congenital Malformations REMACOR, Department of Public Health and Research Support University Hospital of La Réunion Saint Pierre France; ^8^ Center for Clinical Investigation (CIC) 14 10 Clinical Epidemiology, Department of Public Health and Research Support University Hospital of La Réunion, National Institute of Health and Medical Research INSERM Saint‐Denis France; ^9^ Rare Diseases Research Unit Foundation for the Promotion of Health and Biomedical Research in the Valencian Region Valencia Spain; ^10^ REMALAN, Department of Pediatric Surgery CHU de Martinique Fort de France France; ^11^ Directorate for Health Information and Research Tal‐Pietà Malta; ^12^ Department of Medical Genetics, Medical University Pleven Bulgaria; ^13^ Department of Medical Genetics, Poznan University of Medical Sciences Poznan Poland; ^14^ Department of Public Health HSE‐SW St Finbarrs' Hospital Cork Ireland; ^15^ Centre for Research in Epidemiology and Statistics, Obstetrical Perinatal and Pediatric Life Course Epidemiology Research Team (OPPaLE), F‐75004 Université Paris Cité and Université Sorbonne Paris Nord, INSERM, INRAE Paris France; ^16^ Auvergne Registry of Congenital Anomalies (CEMC‐Auvergne), Department of Clinical Genetics, Centre de Référence Maladies Rares “Anomalies du développement et Syndromes Malformatifs”, University Hospital of Clermont‐Ferrand Clermont‐Ferrand France; ^17^ Department of Clinical and Evaluative Epidemiology, Healthcare Trust of the Autonomous Province of Trento, APSS Trento Italy; ^18^ Malformation Monitoring Centre Saxony‐Anhalt, Medical Faculty Otto‐von‐Guericke University Magdeburg Magdeburg Germany; ^19^ Brittany Registry of Congenital Anomalies, CHU Rennes, University of Rennes, INSERM, EHESP, Irset—UMR 1085 Rennes France; ^20^ Unit of Epidemiology of Rare Diseases and Congenital Anomalies, Institute of Clinical Physiology, National Research Council Pisa Italy; ^21^ Ultrasound and Fetal Medicine Unit Woman‐Mother‐Child Department, University Hospital Center CHUV Lausanne Switzerland; ^22^ Eurocat Hainaut‐Namur Center for Human Genetics, Institut de Pathologie et de Génétique Charleroi Belgium; ^23^ OMNI‐Net Programs Rivne Ukraine; ^24^ University Hospitals Southampton Southampton UK; ^25^ School of Health and Medical Sciences City St George's, University of London London UK

**Keywords:** birth defects, heart defects, neural tube defects, orofacial clefts, syndrome, trends

## Abstract

**Background:**

Surveillance of congenital anomaly prevalence over time can identify new teratogens. Anomalies with a genetic cause are excluded from the monitoring.

**Objectives:**

We examined temporal changes in the proportion of genetic diagnoses among cases with a congenital anomaly.

**Methods:**

Data was used from twenty EUROCAT congenital anomaly registries over the birth years 2013 and 2022. All pregnancy outcomes were included. Multilevel binomial regression models were fitted to estimate the annual change in the proportion of genetic diagnoses of all anomalies by registry. Results were additionally reported, excluding cases with trisomy 13, 18, or 21.

**Results:**

Overall, 20% of the 100,099 cases in the study had a genetic diagnosis, and this proportion increased annually by 1.4% (95% CI, 0.8%–1.9%); an absolute increase of approximately 3% from 2013 to 2022. After excluding the trisomies, the overall proportion was 10% with an annual increase of 1.2% (95% CI 0.4%–2.0%). There was considerable variation in the proportion of genetic cases per registry. An increasing proportion of genetic diagnoses was found for five congenital anomaly groups, after excluding the trisomies. We hypothesise that the increase in genetic diagnoses is due to increased access to clinical genetic services, more extensive genetic testing, and the identification of new genes as causes of congenital anomalies.

**Conclusions:**

The modest increase in genetic diagnoses among cases with a congenital anomaly is not expected to have a large impact on the surveillance of the non‐genetic anomalies in the EUROCAT network. EUROCAT will continue to monitor the proportion of genetic diagnoses every five years.

## 1 Background

Congenital anomalies occur in 2%–3% of births and have a large impact on child morbidity and mortality [1, 2, 3]. The cause of congenital anomalies remains unknown in approximately 80% of cases and is thought to be of multifactorial origin [4]. However, for some congenital anomaly cases, a genetic cause can be identified [5, 6]. The chances of finding a genetic cause are dependent on the type of congenital anomaly. For example, in isolated ventricular septal defect (VSD), a genetic cause is rarely found (and hence generally not looked for), whereas in cases with anophthalmos, omphalocele or multiple congenital anomalies, the probability of finding a genetic cause is much higher [6, 7].

The primary objective of the EUROCAT network is to conduct surveillance of congenital anomalies in Europe for the early identification of new teratogens [8]. Hence, when monitoring congenital anomaly prevalence both geographically and over time in order to identify new teratogenic exposures, cases with a known genetic cause are excluded from the analysis [9]. The annual statistical monitoring has found both increasing and decreasing pan‐European trends for specific congenital anomalies [10]. The decreasing trends may be due to an increasing proportion of congenital anomaly cases with a genetic diagnosis, especially in recent years, due to advancements in clinical genetic diagnostics (including non‐invasive prenatal testing, NIPT). An increase in trisomies related to increased maternal age could also play a role. In addition, geographic differences in prevalence may be explained by the proportion of genetic cases differing between European regions, due to variations in access to clinical genetic services and advanced genetic testing (whole exome or genome sequencing has a higher diagnostic yield compared to standard genetic testing) [11].

When performing surveillance of congenital anomalies, it is therefore essential to assess the proportion of genetic diagnoses among cases with a congenital anomaly, to investigate whether this proportion has changed over time, and to examine how it varies geographically. We therefore investigated the proportion of genetic diagnoses among cases with a congenital anomaly using data from twenty EUROCAT registries over the birth years 2013 to 2022.

## 2 Methods

### 2.1 EUROCAT Network

EUROCAT is a network of population‐based congenital anomaly registries that covers nearly 1.5 million births per year, over 25% of all births in Europe. The high‐quality registries actively ascertain congenital anomalies from multiple sources occurring in all birth outcomes (live births, foetal deaths from gestational age 20 weeks and terminations of pregnancy for foetal anomalies at any gestational age (TOPFAs)) [12]. Eight of the registries included in this study report cases diagnosed up to one year of age, whereas two registries have a shorter follow‐up, and the other ten registries have a longer follow‐up [13]. Some registries will update the results of genetic diagnoses after the standard follow‐up time. Information about maximum age at diagnosis, ascertainment sources, genetic data sources and total congenital anomaly prevalence per registry is presented in Table S1. All full member registries provide data on core variables to the Joint Research Center–EUROCAT Central Registry based in Ispra, Italy, since 2015 [8]. Annual statistical surveillance is undertaken to identify if there are new trends in the prevalence of any congenital anomaly and if so whether the trends could be due to primary prevention measures, a new teratogen exposure, or other clinical/administrative reasons such as the adoption of a new diagnostic or screening test.

EUROCAT registries provide data on major congenital anomalies using the International Classification of Diseases version 10 British Paediatric Association extension (ICD‐10/BPA) codes. Coding of congenital anomalies and genetic disorders is done in a highly standardised fashion among all EUROCAT registries, with registries adhering to EUROCAT guide 1.5 and the EUROCAT syndrome guide [12, 14]. A major congenital anomaly is defined as a structural change that has significant medical, social, or cosmetic consequences for the affected individual [15]. EUROCAT has defined a set of homogeneous subgroups of congenital anomalies for surveillance [16]. As a case may have several different major anomalies, they may be in several different anomaly groups. EUROCAT defines a genetic disorder case to include all monogenic disorders (e.g., genetic syndromes, hereditary skin disorders, skeletal dysplasias) and chromosomal anomalies. The following ICD10/BPA codes are included in the genetic disorder group: D821, Q4471, Q6190, Q7402, Q7484, Q751, Q754, Q7581, Q77, Q780‐ Q789, Q796, Q800‐Q824, Q8282, Q8283, Q850, Q851, Q8581, Q87, Q8934, Q90‐ Q93 and Q96‐Q99.

### 2.2 Study Population

Twenty EUROCAT registries from fourteen European countries provided data for the annual EUROCAT statistical surveillance on all cases and birth outcomes from January 2013 to December 2022. Aggregate data were available on the number of cases in each anomaly subgroup by birth year, including the numbers with and without a genetic diagnosis. Cases with multiple congenital anomalies were counted in all relevant subgroups (e.g., both in the VSD and the hypospadias subgroup). Anomaly subgroups in which, by definition, all cases had a genetic disorder (such as chromosomal anomalies and genetic syndromes) were not analysed further. As the prevalence of trisomies 21, 18 and 13 in a region is related to the level of prenatal screening (first trimester or later) and the maternal age distribution [17], and as these may change over time, results are reported with and without these trisomies.

### 2.3 Statistical Methods

Multilevel binomial regression models were fitted to estimate the annual change in the proportion of genetic diagnoses of all anomalies by registry, expressed as a proportional change, assuming a constant rate of change in the trend analysis using STATA v16. In addition, the annual changes in the proportion of genetic diagnoses by anomaly subgroup were estimated. Data were aggregated over 2‐year periods to plot figures that were not too greatly influenced by sampling errors due to small numbers of cases.

### 2.4 Missing Data

For a case to be included in a EUROCAT registry, it must have a congenital anomaly and a year of birth. For a case to be included in the EUROCAT central database, it needs to have a code for a major congenital anomaly and a year of birth. The genetic cases are identified according to the ICD‐10/BPA codes for genetic disorders. Therefore, no data were missing in this analysis.

## 3 Results

**FIGURE 1:**
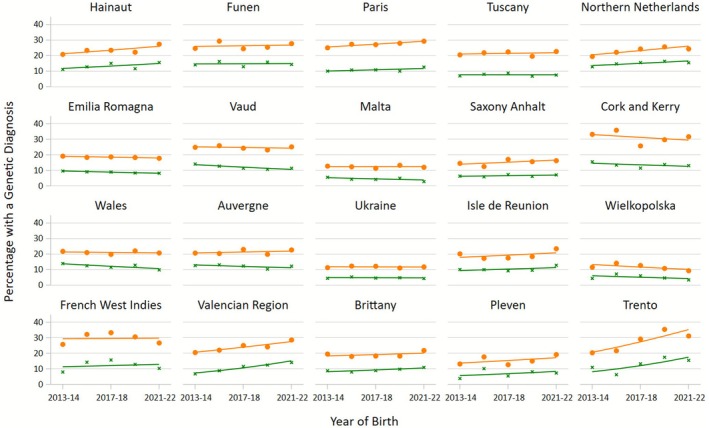
Percentage of genetic diagnoses by registry over 2013–2022 including trisomies 13, 18 and 21 (orange) and excluding trisomies 13, 18 and 21 (green).

Overall, 20% (95% CI 20, 21%) of all 100,099 cases in the study had a genetic diagnosis, and 10% (95% CI 9, 10%) after excluding the three trisomies (Table 1). On a pan‐European level, the proportion of cases with a genetic diagnosis increased from 2013 to 2022, with an annual percentage change of 1.4% (95% CI, 0.8%–1.9%), including cases of trisomies. This means that if the proportion of genetic diagnoses is 20% in 1 year, then the next year it will be 20.3% (0.20 × 1.014), which is approximately an additional 0.28% per year or an absolute increase of 3% (0.20 × (1.014^9^−1)) over the whole 10‐year period. When excluding the trisomies, the annual percentage change was 1.2% (95% CI 0.4%–2.0%), which is approximately an additional 0.12% per year or an absolute increase of 1.1% over 10 years. The proportion of genetic cases reported by each registry varied from 12% in Malta, Ukraine and Wielkopolska up to 31% in Cork and Kerry (Table 1). Fifteen of the twenty registries reported an increasing proportion of genetic cases over time, although the increases were often modest (Table 1 and Figure 1). One registry, Wielkopolska, reported a clearly decreasing trend. After the exclusion of the trisomies, the proportion of genetic diagnoses varied between 5% in Malta, Ukraine and Wielkopolska to 15% in Funen and Northern Netherlands. After excluding the trisomies, eleven registries reported an increasing trend. In addition, two registries reported a clearly decreasing trend (Wales and Wielkopolska). Registries with a higher proportion of cases diagnosed after 1 year of life (giving time for more genetic diagnoses to be completed and reported) tended to have a higher overall proportion of genetic diagnoses excluding the trisomies (Figure S1).

**TABLE 1:** Percentage of genetic diagnoses by EUROCAT registry over birth years 2013 to 2022.

	Genetic diagnoses include trisomies			All cases of trisomy have been excluded		
	Number of cases	Genetic diagnoses 2013–2022	Annual percentage change in genetic diagnoses	Number of cases	Genetic diagnoses 2013–2022	Annual percentage change in genetic diagnoses
		Percentage (95% CI)	Percentage (95% CI)		Percentage (95% CI)	Percentage (95% CI)
Hainaut‐Namur (Belgium)	2512	24 (22, 25)	3.6 (1.0, 6.2)	2215	13 (12, 15)	3.5 (−0.8, 8.0)
Funen (Denmark)	1249	26 (24, 29)	0.3 (−3.9, 4.6)	1079	15 (13, 17)	0 (−5.7, 6.0)
Paris (France)	7822	27 (26, 28)	2.4 (0.6, 4.3)	6378	11 (10, 12)	2.4 (−0.4, 5.3)
Tuscany (Italy)	5664	21 (20, 23)	0.9 (−1.5, 3.4)	4819	8 (7, 8)	0.3 (−3.2, 4.0)
Northern Netherlands	4789	23 (22, 24)	4.5 (−4.3, 14.2)	4328	15 (14, 16)	3.4 (0.1, 6.7)
Emilia Romagna (Italy)	9178	19 (18, 19)	−0.7 (−1.8, 0.5)	8208	9 (8, 10)	−2 (−5.0, 1.1)
Vaud (Switzerland)	2695	25 (23, 26)	−0.6 (−3.0, 1.8)	2309	12 (11, 14)	−3.7 (−7.6, 0.3)
Malta	1181	12 (11, 14)	0.3 (−6.1, 7.3)	1084	5 (3, 6)	−3.8 (−14.0, 7.6)
Saxony Anhalt (Germany)	4918	15 (14, 16)	2.6 (−0.3, 5.7)	4463	7 (6, 7)	1.6 (−1.7, 5.0)
Cork and Kerry (Ireland)	1981	31 (29, 33)	−1.7 (−4.9, 1.6)	1575	14 (12, 15)	−1.9 (−6.5, 2.9)
Wales (UK)	9493	21 (20, 22)	−0.3 (−2.1, 1.5)	8545	12 (12, 13)	−3.7 (−6.0, −1.5)
Auvergne (France)	3962	21 (20, 23)	0.7 (−9.0, 11.6)	3553	12 (11, 13)	−2.3 (−6.2, 1.8)
OMNI‐net (Ukraine)	6060	12 (11, 13)	0.2 (−1.7, 2.1)	5615	5 (4, 5)	−0.5 (−4.8, 4.1)
Isle de Reunion (France)	4710	19 (18, 20)	2.4 (0.2, 4.7)	4238	10 (9, 11)	2.5 (−1.1, 6.2)
Wielkopolska (Poland)	8329	12 (11, 12)	−3.4 (−5.7, −1.2)	7755	5 (5, 6)	−4 (−7.3, −0.6)
French West Indies (France)	2220	30 (28, 32)	0.2 (−2.8, 3.3)	1778	12 (11, 14)	1.8 (−2.9, 6.7)
Valencian Region (Spain)	9463	23 (23, 24)	5 (3.1, 7.0)	8069	10 (10, 11)	11.1 (8.0, 14.4)
Brittany (France)	12,128	19 (18, 20)	2 (0.2, 3.8)	10,809	9 (9, 10)	4.3 (1.9, 6.7)
Pleven (Bulgaria)	553	15 (12, 18)	4.1 (−4.2, 13.2)	503	7 (5, 9)	5.2 (−6.9, 18.8)
Trento (Italy)	1192	28 (25, 30)	9.5 (4.0, 15.2)	982	12 (10, 15)	11.8 (4.3, 19.8)
Total	100,099	20 (20, 21)	1.4 (0.8, 1.9)	88,305	10 (9, 10)	1.2 (0.4, 2.0)

Abbreviation: CI, confidence interval.

The proportions of genetic diagnoses varied considerably according to the anomaly subgroups. High proportions of genetic diagnoses were found in atrioventricular septal defect (AVSD) (61%), arhinencephaly/holoprosencephaly (45%) and omphalocele (42%) (Table 2). Low proportions of genetic diagnoses were found for congenital pulmonary airway malformations (2%), atresia of bile ducts (3%), gastroschisis (3%), posterior urethral valves (2%), hypospadias (2%), hip dislocation (2%), conjoined twins (0%), VACTERL association (3%) and caudal regression sequence (2%). After exclusion of the trisomies, the highest proportions of genetic diagnoses were found in aortic atresia/interrupted aortic arch (26%), common arterial truncus (25%) and arhinencephaly/holoprosencephaly (22%). Some anomalies, such as AVSD and duodenal atresia/stenosis (and to a lesser extent, persistent ductus arteriosus, Hirschsprung's disease and omphalocele), were primarily observed in cases with trisomies. On the contrary, in glaucoma, several heart defects, atresia of bile ducts, hypospadias and Pierre‐Robin sequence, almost exclusively non‐trisomy genetic diagnoses were made.

**TABLE 2:** Percentage of genetic diagnoses in congenital anomaly cases born in 2013–2022 and annual percentage change from 2013 to 2022 per anomaly group.

Anomaly^a^	Genetic diagnoses include trisomies			All cases of trisomy have been excluded		
	*n* cases 2013–2022	Genetic diagnoses 2013–2022	Annual percentage change in genetic diagnoses	*n* cases 2013–2022	Genetic diagnoses 2013–2022	Annual percentage change in genetic diagnoses
		% (95% CI)	% (95% CI)		% (95% CI)	% (95% CI)
Neural tube defects	3493	7 (6, 8)	−1.5 (−6.0, 3.1)	3372	4 (3, 4)	−0.2 (−6.3, 6.3)
Anencephaly and similar	1341	4 (3, 5)	−1.1 (−10.3, 9.2)	1305	1 (1, 2)	10.9 (−6.1, 30.8)
Encephalocele and meningocele	440	13 (10, 16)	0.9 (−8.9, 11.8)	423	9 (7, 12)	−1.5 (−12.5, 10.8)
Spina bifida	1712	8 (7, 9)	−1.8 (−7.7, 4.5)	1644	4 (3, 5)	−0.6 (−8.8, 8.3)
Hydrocephaly	1845	17 (16, 19)	4.4 (−0.1, 9.1)	1751	13 (11, 14)	6.7 (1.4, 12.4)
Severe microcephaly	1159	21 (19, 24)	2.7 (−2.6, 8.2)	1117	18 (16, 20)	2.5 (−3.2, 8.5)
Arhinencephaly/holoprosencephaly	623	45 (41, 49)	6.7 (0.1, 13.7)	437	22 (18, 26)	8 (−1.5, 18.3)
Agenesis of corpus callosum	1039	20 (18, 22)	−0.8 (−6.3, 5.0)	988	16 (14, 18)	−2.3 (−8.4, 4.2)
Anophthalmos/microphthalmos	405	30 (26, 35)	3.7 (−4.2, 12.2)	353	20 (16, 25)	5.7 (−4.1, 16.5)
Anophthalmos	70	14 (7, 25)	17.2 (−7.9, 49.2)	65	8 (3, 17)	19.9 (−14.1, 67.3)
Congenital cataract	583	12 (9, 15)	6.4 (−2.8, 16.5)	562	9 (6, 11)	7.2 (−3.6, 19.3)
Congenital glaucoma	151	13 (8, 20)	11.9 (−7.6, 35.4)	150	13 (8, 19)	16.2 (−4.8, 41.8)
Anotia and atresia/stenosis/stricture of external auditory canal	380	15 (12, 19)	−4.9 (−14.6, 5.9)	363	11 (8, 15)	−6.2 (−17.2, 6.3)
Severe congenital heart defects	8749	22 (21, 23)	1.8 (−0.0, 3.7)	7487	9 (8, 10)	4.6 (1.6, 7.7)
Common arterial truncus	260	28 (22, 34)	2.7 (−8.1, 14.8)	250	25 (20, 31)	2.5 (−8.9, 15.2)
Double outlet right ventricle	553	20 (17, 23)	0.1 (−7.5, 8.3)	501	12 (9, 15)	4.1 (−6.0, 15.2)
Complete transposition of great arteries (D‐TGA)	1169	5 (3, 6)	6.2 (−4.2, 17.7)	1150	3 (2, 4)	7.7 (−5.2, 22.3)
Single ventricle	283	15 (11, 20)	9.8 (−2.8, 24.0)	259	7 (4, 11)	14.5 (−3.5, 35.9)
Corrected transposition of great arteries (L‐TGA)	95	11 (5, 19)	49.2 (−0.5, 123.6)	89	4 (1, 11)	16.6 (−29.8, 93.7)
Ventricular septal defect (VSD)	16,588	10 (10, 11)	0.8 (−1.0, 2.7)	15,565	4 (4, 4)	0.9 (−1.9, 3.8)
Atrial septal defect (ASD)	5320	16 (15, 17)	2.9 (0.2, 5.8)	4797	7 (6, 8)	2.3 (−1.8, 6.6)
Atrioventricular septal defect (AVSD)	1707	61 (59, 64)	2.2 (−1.4, 5.9)	767	14 (12, 17)	6.2 (−1.6, 14.7)
Tetralogy and pentalogy of Fallot	1227	21 (19, 24)	0.7 (−4.3, 5.9)	1097	12 (10, 14)	5.7 (−1.2, 13.0)
Triscuspid atresia and stenosis	250	9 (6, 13)	11.3 (−5.4, 30.9)	241	5 (3, 9)	18.2 (−5.1, 47.4)
Ebstein's anomaly	188	11 (7, 16)	−4.9 (−20.3, 13.6)	179	6 (3, 11)	4.7 (−16.4, 31.2)
Pulmonary valve stenosis	1644	11 (10, 13)	3.0 (−2.7, 9.0)	1609	9 (8, 11)	5 (−1.5, 11.8)
Pulmonary valve atresia	415	13 (10, 17)	−0.3 (−10.7, 11.3)	403	11 (8, 14)	−2.3 (−13.7, 10.6)
Aortic valve atresia/stenosis	534	8 (6, 10)	−3.4 (−13.6, 7.8)	527	7 (5, 9)	−2.6 (−13.9, 10.0)
Mitral valve atresia/stenosis	142	10 (5, 16)	14.1 (−10.3, 45.1)	138	7 (4, 13)	9 (−17.2, 43.5)
Hypoplastic left heart (HLH/HLHS)	892	13 (11, 15)	5.7 (−1.6, 13.6)	844	8 (6, 10)	10.5 (0.7, 21.2)
Hypoplastic right heart (HRH/HRHS)	258	10 (7, 15)	15.2 (−2.5, 36.1)	247	6 (4, 10)	9.8 (−10.8, 35.1)
Coarctation of aorta	1238	9 (7, 10)	3.8 (−3.5, 11.6)	1206	6 (5, 8)	8.4 (−0.6, 18.2)
Aortic atresia/interrupted aortic arch	170	30 (23, 37)	−2.6 (−14.5, 10.9)	160	26 (19, 33)	−1.3 (−14.5, 13.9)
Total anomalous pulmonary venous return	227	7 (4, 12)	−6.2 (−22.5, 13.6)	225	7 (4, 11)	−0.5 (−18.8, 22.0)
Patent ductus arteriosus (PDA) as only CHD in term infants	1106	12 (11, 14)	3.3 (−3.6, 10.7)	1012	4 (3, 6)	8.6 (−3.5, 22.2)
Congenital pulmonary airway malformations	343	2 (1, 5)	−2.2 (−23.6, 25.2)	340	1 (0, 3)	8.6 (−21.2, 49.8)
Cleft lip with or without cleft palate	2923	12 (11, 13)	2.5 (−1.6, 6.8)	2733	6 (5, 7)	8.4 (2.3, 14.7)
Cleft palate	2026	17 (15, 18)	4.2 (−0.2, 8.7)	1980	15 (13, 16)	4.3 (−0.3, 9.2)
Oesophageal atresia	926	12 (10, 14)	2.3 (−4.7, 9.7)	868	6 (5, 8)	11.2 (0.5, 23.1)
Duodenal atresia or stenosis	463	29 (25, 34)	−4.2 (−10.9, 3.1)	346	5 (3, 8)	6.2 (−11.0, 26.7)
Atresia or stenosis of other parts of small intestine	305	5 (3, 8)	−10.2 (−25.9, 8.9)	300	4 (2, 6)	−15 (−33.0, 7.7)
Ano‐rectal atresia or stenosis	1157	11 (9, 13)	1.1 (−5.3, 7.9)	1116	8 (6, 9)	3.9 (−3.8, 12.3)
Hirschsprung's disease	481	12 (10, 16)	7.9 (−2.7, 19.6)	437	4 (2, 6)	6.5 (−11.6, 28.3)
Atresia of bile ducts	159	3 (1, 7)	−9.4 (−35.5, 27.1)	158	3 (1, 6)	−9.6 (−37.1, 29.7)
Annular pancreas	84	27 (18, 38)	−3.8 (−20.6, 16.6)	68	10 (4, 20)	15.7 (−19.4, 66.1)
Anomalies of intestinal fixation	548	19 (16, 22)	−0.3 (−8.1, 8.3)	515	14 (11, 17)	−1.4 (−10.5, 8.5)
Diaphragmatic hernia	1007	13 (11, 15)	6.0 (−1.1, 13.5)	957	8 (6, 10)	1.8 (−6.6, 10.9)
Gastroschisis	672	3 (2, 5)	17.4 (−0.1, 38.0)	661	2 (1, 3)	1 (−19.0, 25.9)
Omphalocele	1276	42 (40, 45)	4.4 (0.0, 9.0)	858	14 (12, 17)	1.6 (−5.4, 9.1)
Unilateral renal agenesis	1803	5 (4, 7)	3.7 (−3.7, 11.7)	1776	4 (3, 5)	6.4 (−2.4, 16.0)
Bilateral renal agenesis including Potter sequence	412	9 (6, 12)	2.2 (−10.2, 16.3)	410	8 (6, 11)	3.2 (−9.6, 17.9)
Multicystic renal dysplasia	1762	7 (6, 8)	7.3 (0.3, 14.8)	1745	6 (5, 7)	5.2 (−2.1, 13.1)
Congenital hydronephrosis including ureter obstruction	6438	4 (4, 5)	2.7 (−1.8, 7.4)	6337	3 (2, 3)	3.9 (−1.8, 9.9)
Lobulated, fused and horseshoe kidney and ectopic kidney	1555	10 (9, 12)	−1.4 (−7.1, 4.6)	1486	6 (5, 8)	0.1 (−7.3, 8.1)
Bladder exstrophy and/or epispadias	221	4 (2, 7)	1.3 (−22.1, 31.8)	218	2 (1, 5)	14.4 (−19.9, 63.5)
Posterior urethral valves	508	2 (1, 4)	19.0 (−6.5, 51.3)	502	1 (0, 2)	13.4 (−18.0, 56.9)
Hypospadias	7127	2 (2, 3)	−0.2 (−5.6, 5.4)	7107	2 (2, 3)	−1 (−6.6, 5.0)
Limb reduction defects (LRD)	1976	18 (16, 20)	−1.0 (−5.0, 3.2)	1836	12 (10, 13)	−0.6 (−5.6, 4.6)
Transverse LRD	226	6 (3, 10)	16.7 (−5.8, 44.4)	221	4 (2, 7)	8.7 (−16.1, 40.9)
Longitudinal preaxial LRD	563	29 (26, 33)	−1.9 (−8.4, 5.2)	463	14 (11, 18)	−3 (−11.9, 6.8)
Longitudinal postaxial LRD	167	12 (7, 18)	2.9 (−14.3, 23.6)	163	10 (6, 15)	0.7 (−17.1, 22.3)
Longitudinal central LRD	148	22 (15, 29)	5.8 (−8.7, 22.7)	143	19 (13, 26)	10.3 (−6.0, 29.4)
Club foot—talipes equinovarus	4372	11 (10, 12)	4.5 (1.1, 8.1)	4160	7 (6, 7)	3.9 (−0.5, 8.6)
Hip dislocation	2597	2 (1, 2)	−5.3 (−15.4, 5.9)	2596	2 (1, 2)	−6.4 (−16.4, 4.9)
Polydactyly	4706	9 (8, 9)	−0.8 (−4.4, 3.0)	4490	4 (4, 5)	1.3 (−4.0, 6.8)
Syndactyly	1611	18 (17, 20)	3.4 (−1.3, 8.3)	1545	15 (13, 17)	1.6 (−3.5, 7.0)
Craniosynostosis	1344	11 (9, 13)	5.6 (−1.1, 12.8)	1324	9 (8, 11)	6.4 (−0.8, 14.1)
Congenital constriction bands/amniotic band sequence	171	4 (2, 8)	−10.0 (−32.2, 19.5)	170	4 (1, 8)	−10 (−33.9, 22.6)
Situs inversus	327	12 (9, 16)	8.7 (−3.8, 22.9)	321	10 (7, 14)	9.3 (−4.3, 24.8)
Conjoined twins	50	0 (0, 7)	NA	50	0 (0, 7)	NA
VATER/VACTERL association	217	3 (1, 6)	−2.5 (−28.8, 33.5)	217	3 (1, 6)	−2.5 (−28.4, 32.9)
Pierre‐Robin sequence	486	14 (11, 18)	6.8 (−2.5, 17.0)	478	13 (10, 16)	9.1 (−0.9, 20.0)
Caudal regression sequence	84	2 (0, 8)	−8.8 (−51.9, 72.9)	83	1 (0, 7)	18.9 (−52.1, 195.2)
Septo‐optic dysplasia	58	7 (2, 17)	−11.4 (−40.3, 31.7)	57	5 (1, 15)	−18.2 (−50.1, 33.9)

*Note:* All results not adjusted for registry due to small numbers in some registries.

Abbreviation: CI, confidence interval.

^a^ Only subgroups with 50 cases or more over 2013–2022 are shown. The classification of anomaly subgroups is not mutually exclusive (e.g., a case with VSD and hypospadias is counted in both the VSD and the hypospadias group).

It is essential to note that the proportion of genetic diagnoses in Table 2 applies to all cases with a specific anomaly. For example, a case with an isolated VSD is included in the VSD group, but also a case with a VSD and another heart defect or a VSD and an anomaly in another organ system is included. Ideally, we would like to show the proportion of genetic diagnoses for all isolated anomalies; however, this would greatly reduce the numbers. Therefore, we only calculated the proportion of genetic diagnoses in the largest subgroup, which is VSD. We observed that this reduces the proportion of genetic diagnoses (including the trisomies) from 10% in all cases with a VSD to 5% in cases with an isolated VSD. After exclusion of the trisomies, the proportion of genetic diagnoses decreased from 4% in all VSD cases to 1% in isolated VSD cases. This indicates that if there are additional anomalies, the case is more likely to have a genetic diagnosis.

**FIGURE 2:**
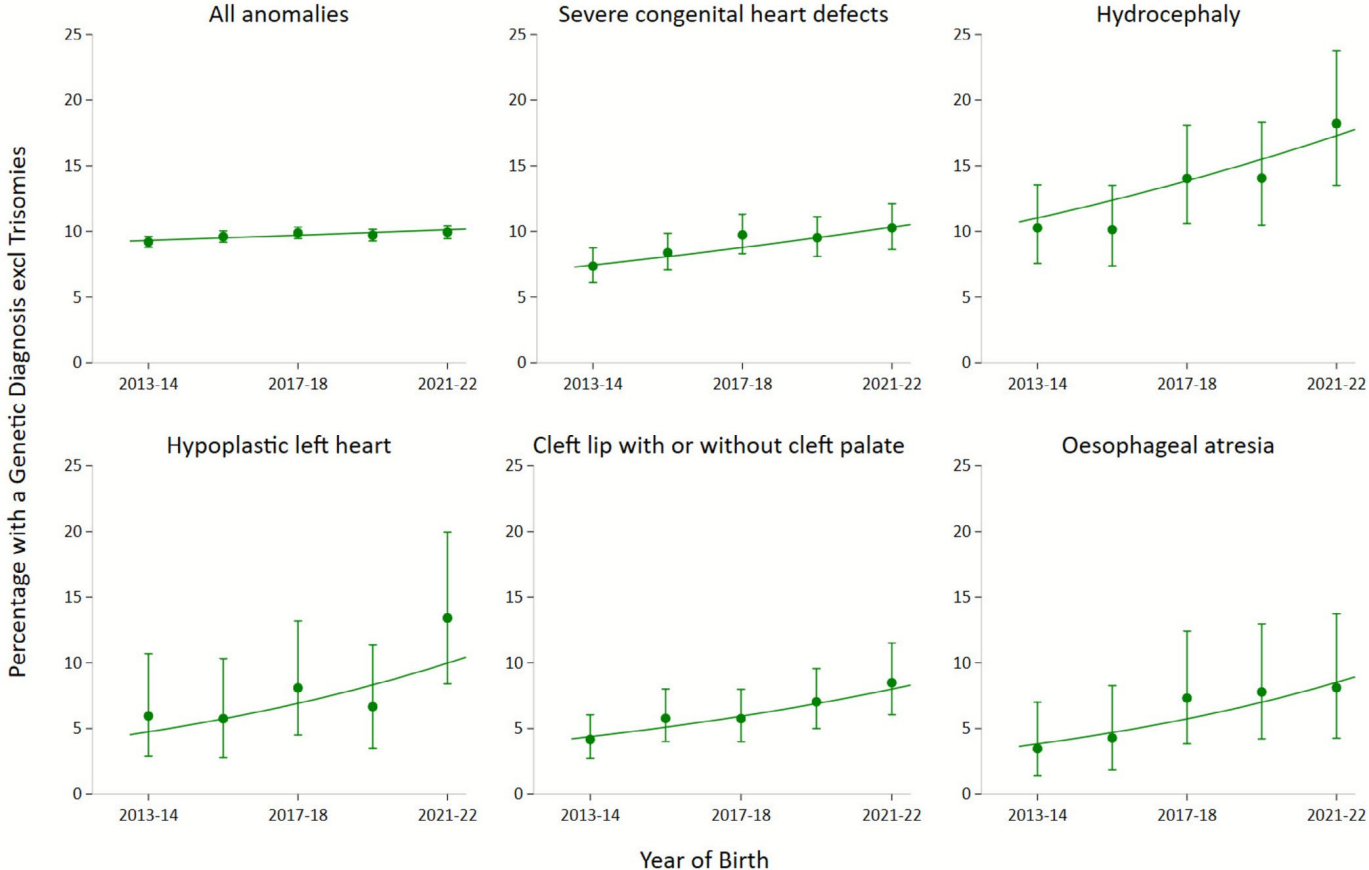
Anomalies with clear trends in the percentage of genetic diagnoses over 2013–2022, excluding trisomies 13, 18 and 21: Biennial estimates and 95% confidence intervals.

We observed an increasing proportion of genetic diagnoses in many congenital anomaly groups over time after excluding the trisomies, which was most clearly seen in the following five anomaly groups: hydrocephaly, severe congenital heart defects, hypoplastic left heart, cleft lip with or without cleft palate and oesophageal atresia with or without tracheo–oesophageal fistula (Table 2). Figure 2 shows the time trends for these five anomaly groups over 2013–2022.

## 4 Comment

### 4.1 Principal Findings

Our European multi‐centre population‐based study showed a generally modest increase in the proportion of genetic diagnoses among cases with major congenital anomalies over a 10‐year period. This increase was present both when including and excluding the three most common trisomies. We also found that there was considerable variation in the proportion of genetic cases per registry. Our study also showed a clearly increasing proportion of genetic diagnoses over 2013 and 2022 for five specific congenital anomaly groups, after excluding the trisomies.

### 4.2 Strengths of the Study

A strength of this study is that we report on recent data of a large number of congenital anomaly cases. Also, we used highly standardised congenital anomaly data from the EUROCAT network, making it possible to combine data from registries in different European countries.

### 4.3 Limitations of the Data

Other factors that influence the proportion of genetic diagnoses will, however, vary between countries and registries. For example, access to clinical genetic diagnostic care and the implementation of new genetic tests and prenatal screening programmes will vary among countries and the available genetic data sources and the length of follow‐up of liveborn congenital anomaly cases vary between registries. This heterogeneity will have resulted in an underestimation of the proportion of congenital anomalies with a genetic cause.

When studying the increase in the proportion of cases with a genetic diagnosis, it is important to note that genetic diagnoses are sometimes made later in life and might not have been identified yet in cases born in more recent years [18]. If a genetic diagnosis is made, some registries will update this in the central JRC‐EUROCAT database, but the length of follow‐up of cases differs between registries. In this study, registries with a higher proportion of cases diagnosed after 1 year of age had a higher proportion of genetic diagnoses after excluding the trisomies (as trisomies are usually diagnosed early in life). In addition, when examining the proportion of genetic diagnoses among cases with a congenital anomaly, it is essential to consider that the ascertainment methods of registries differ; a registry with excellent ascertainment may include more cases with mild anomalies, which are not often genetic. In this study, we were only able to present the proportion of genetic diagnoses for isolated VSD and not for other isolated anomalies, because the classification of isolated anomalies in EUROCAT includes cases with anomalies in the same organ system. Also, EUROCAT does not have sufficient data on genetic testing to be able to report which percentage of (specific) congenital anomaly cases underwent genetic testing. Another limitation is that there might be some overreporting of minor array results with codes Q935, Q936 or Q923 in the EUROCAT database. Cases with these codes were classified as genetic, but the deletion or duplication might not have clinical consequences in all cases. There may be differences across registries and over time in the reporting of minor array results.

### 4.4 Interpretation

The increasing proportion of genetic diagnoses may be explained by several factors. It is likely that the number of genetic analyses is increasing and that genetic testing has become more extensive over the study period. In some countries, access to clinical genetic diagnostic care might have improved over time. Registries may also have improved their data sources to have more information from genetic departments and in this way increased their proportion of genetic cases. As prenatal screening for trisomy 21, 13 and 18 by non‐invasive prenatal testing (NIPT) is nowadays done earlier and is more widespread [19], this is expected to have quite an impact on the overall proportion of genetic diagnoses. In addition, increased maternal age will lead to an increase in trisomies [17], and therefore, we have also given results for the proportion of genetic cases without the three most common trisomies.

We found major differences in the proportion of genetic cases across registries, with a rather low proportion in the two Eastern European registries (Ukraine, Wielkopolska) and Malta, which are also the registries where terminations of pregnancy for foetal anomalies are illegal or only legal under specific circumstances. The low proportion of genetic cases may be explained by the limited availability and accessibility of genetic testing in these countries, or a more cautious attitude towards the personal benefits of genetic testing among Eastern European citizens [20]. Being religious was found to be associated with a more negative opinion on genetic testing [21]. Also, when costs for advanced genetic testing are not reimbursed, like in Poland, this will be a barrier to performing genetic tests [22, 23]. Another possible reason for the low proportion of genetic diagnoses in these countries might be that their ascertainment of genetic diagnoses is lower, due to fewer genetic data sources or a shorter follow‐up of cases; however, both seem comparable to those of other registries.

The proportion of genetic diagnoses differed between the different anomaly groups. As expected, anomalies that are frequent in trisomy 21 (e.g., AVSD and duodenal atresia), trisomy 18 (heart defects and orofacial clefts) and trisomy 13 (holoprosencephaly, omphalocele, anophthalmos/microphthalmos and heart defects) had a high percentage of genetic diagnoses. Arhinencephaly/holoprosencephaly also had a high percentage of genetic diagnoses after the exclusion of the trisomies (22%). Possibly triploidy, other chromosomal aberrations and mutations in genes like sonic hedgehog are responsible for this [24]. The highest proportions of non‐trisomy genetic diagnoses were found in aortic atresia/interrupted aortic arch (26%) and common arterial truncus (25%), which are frequently occurring in 22q11.2 deletion syndrome [25, 26].

An increasing trend was seen in the proportion of non‐trisomy genetic diagnoses for five congenital anomaly groups. For severe congenital heart defects, the proportion of non‐trisomy genetic diagnoses increased to 10.3% in 2022 in our study and up to 13.4% for hypoplastic left heart. Heart defects can occur in Turner syndrome, 22q11.2 deletion syndrome, other cytogenetic anomalies and mutations in several genes (e.g., CHARGE syndrome) [27]. In the Netherlands, in euploid foetuses with severe CHD born between 2012 and 2016, a definite genetic diagnosis was found in 13% (94/708) [26], which is comparable to our results. The most common diagnosis was 22q11.2 deletion syndrome. A recently published review found a growing number of genes involved in hydrocephalus and also showed that more extensive genetic testing was performed in hydrocephalus patients over time [28]. This could explain the increased proportion of genetic diagnoses over time that we found. We think that the same explanation might be true for the increase in genetic diagnoses for cleft lip with or without cleft palate [29, 30], and oesophageal atresia. However, the genetic yield in oesophageal atresia is relatively low [31].

## 5 Conclusions

This paper provides an overview of the percentage of genetic diagnoses in European cases with congenital anomalies. This information is useful for counselling and can perhaps aid the planning of genetic testing after an anomaly is detected on a prenatal ultrasound scan. The percentage of genetic diagnoses is different between European countries and is increasing over time. However, the increase is modest and is not expected to have a large impact on the prevalence of non‐genetic anomalies in the EUROCAT network, and is unlikely to explain the decreasing pan‐European trends observed in recent annual statistical surveillance. When interpreting trends in future surveillance of the EUROCAT network, consideration will be given to whether any changes have occurred in the proportions of genetic cases. The proportion of genetic cases will be monitored every 5 years to provide up‐to‐date information to clinicians and parents upon receiving an initial diagnosis of an anomaly.

## Author Contributions

J.K.M. and E.G. conceived and designed the work. A.P. extracted the data from the JRC‐EUROCAT central database and J.K.M. did the statistical analyses. J.E.H.B. and E.G. drafted the report and interpreted the results. A.P., I.B., D.T. and D.W. gave feedback from the start. All other authors acquired data from their registry and gave feedback on the final version of the paper. All authors have approved the final version.

## Ethics Statement

For this study, pseudonymized data from EUROCAT registries were used, all of which have their own ethics approval. Therefore, no specific ethics approval was required for this study.

## Conflicts of Interest

The authors declare no conflicts of interest.

## Supporting information


**Figure S1:** Percentage of genetic diagnoses excluding trisomies 13, 18 and 21 in each registry according to the proportion of cases that are diagnosed after 1 year.

**Table S1:** Information on maximum age at diagnosis, data sources of case ascertainment, genetic data sources and total congenital anomaly prevalence of all participating EUROCAT registries.

## Data Availability

The data analysed during this study belong to the individual EUROCAT registries. Applications to analyse these data will be considered by the JRC‐EUROCAT management committee in consultation with the local registries.
