# Supplementary figures and images for: Genetic Diagnoses Among Congenital Anomaly Cases in Europe: Data From the EUROCAT Network
Source: Paediatr Perinat Epidemiol. 2025 Nov 24;40(3):414–25. doi: 10.1111/ppe.70099 (PMC13124677; doi:10.1111/ppe.70099)

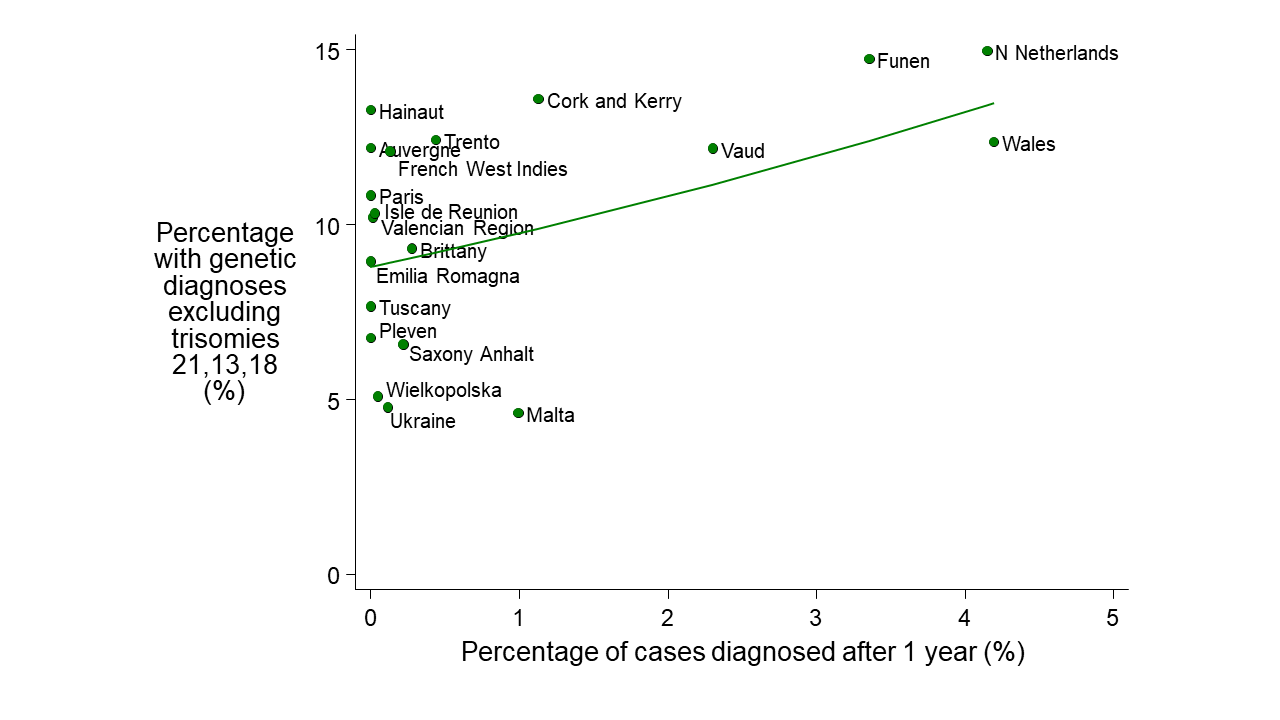

Supplement: Supplementary file 1 — Figure S1: Percentage of genetic diagnoses excluding trisomies 13, 18 and 21 in each registry according to the proportion of cases that are diagnosed after 1 year. [file PPE-40-414-s001.png]
