# Supplementary material for: Genetic Diagnoses Among Congenital Anomaly Cases in Europe: Data From the EUROCAT Network
Source: Paediatr Perinat Epidemiol. 2025 Nov 24;40(3):414–25. doi: 10.1111/ppe.70099 (PMC13124677; doi:10.1111/ppe.70099)
Supplement: Supplementary file 2 — Table S1: Information on maximum age at diagnosis, data sources of case ascertainment, genetic data sources and total congenital anomaly prevalence of all participating EUROCAT registries. [file PPE-40-414-s002.docx]

**Supplementary Table 1** Information on maximum age at diagnosis, data sources of case ascertainment, genetic data sources and total congenital anomaly prevalence of all participating EUROCAT registries

| **Registry** | **Maximum age at diagnosis^a^** | **Data sources of case ascertainment^a^** | **Genetic data sources^a^** | **Total congenital anomaly prevalence compared to EUROCAT average 265.9 [264.2 – 267.7]^b^** |
| --- | --- | --- | --- | --- |
| Hainaut-Namur (Belgium) | 1 year | Prenatal and delivery units, neonatal and paediatric departments, pathological data | All cytogenetic and genetic data from the Institute of Pathology and Genetics of Charleroi | 249.7 [236.6 - 263.2] |
| Funen (Denmark) | 5 years | Obstetric and paediatric departments including the paediatric cardiology centre, post‐mortem examinations | Cytogenetic laboratory. The registry receives an annual list of all abnormal karyotypes diagnosed prenatally and postnatally. | 274.9 [254 - 297.1] |
| Paris (France) | 1 week | Delivery units, paediatric departments, pathology departments, health and birth certificates | Cytogenetic laboratories. The registry systematically (at least once a year) consults the three main cytogenetic laboratories in Parisian hospitals. | 337.9 [327.3 - 348.8] |
| Tuscany (Italy) | 1 year | Obstetrical and maternity units, paediatric departments, paediatric cardiology departments, paediatric surgery units, paediatric cardiac surgery units, prenatal diagnostic centres | Medical genetics units | 249.2 [239.8 - 258.8] |
| Northern Netherlands | 10 years | Obstetric records, hospital administration data, pathology records, paediatric cardiology centre, etc. | Cytogenetic laboratory results are electronically downloaded from the genetics department and include all abnormal karyotype reports, both from prenatal and postnatal samples. For cases reported to the registry it is verified whether any genetic tests were performed, and test results are registered in the database. | 316.1 [303.6 - 329] |
| Emilia Romagna (Italy) | 1 year | Neonatology, paediatrics and obstetrics departments, delivery units, the hospital discharge database, the regional TOPFA database, the rare disease registry, mortality database | Cytogenetics test results and karyotypes where available will be provided to EUROCAT. | 252.7 [244.7 - 260.8] |
| Vaud (Switzerland) | No upper age limit | Delivery units, paediatric departments, pathology units, paediatric cardiology, prenatal echocardiography centres | Cytogenetic laboratories | 296.6 [280.4 - 313.4] |
| Malta | 1 year | Delivery, obstetric and paediatric wards, paediatric echo cardiology records, national mortality register, national obstetric information system database, hospital activity analysis database and rare disease register | Genetic clinic records | 285.9 [264.2 - 308.9] |
| Saxony Anhalt (Germany) | 1 year | Delivery units, paediatric departments, including paediatric cardiology, laboratories, prenatal diagnostic centres, departments of pathology and other specialities | Cytogenetic laboratories. The registry receives some results from cytogenetic laboratories but not through direct access or via electronic transfer. Some are received indirectly via obstetric or paediatric notes. | 288.6 [276.7 - 300.8] |
| Cork and Kerry (Ireland) | 7 years, but syndromes are registered if diagnosed later | Obstetric and neonatal departments, paediatric cardiology and orthopaedics outpatient letters, hospital discharge data (HIPE), birth notifications, stillbirth certificates, Central Statistics Office (CSO) data on deaths in children up to the age of 2 years and post mortem examinations. The paediatric cardiology and cardiac surgery database are historical sources of confirmatory data. | The national cytogenetic laboratory is a historical source of confirmatory data | 251.1 [235.7 - 267.3] |
| Wales (UK) | 1 year,  but all chromosomal anomalies and syndromes are registered if diagnosed later | Antenatal clinics, delivery units, paediatric departments, paediatric cardiology centres, ophthalmology, pathology, orthopaedics, maxillo-facial and regional centres of paediatric surgery | Cytogenetics. The cytogenetic laboratory in Cardiff provides a download of data of all abnormal karyotypes on a quarterly basis. | 271.8 [263.3 - 280.5] |
| Auvergne (France) | 1 year | Paediatric records, pathology laboratory, child health services, midwives, birth notifications, maternity unit records, orthopaedics, cardiology, paediatric surgery, paediatric radiology, department of prenatal diagnosis, multidisciplinary committee of prenatal diagnosis (CPDP) | Genetics laboratories, specialised departments for medical genetics | 337.2 [321 - 354] |
| OMNI-net (Ukraine) | 1 year | Hospital admission/discharge summaries, obstetrics, neonatalogy, paediatrics, paediatric cardiology centres, specialty clinics, laboratories and other services | Cytogenetic laboratories. Our cytogenetic laboratories are the only ones in the region and they provide us with study reports. | 280.6 [269.2 - 292.3] |
| Isle de Reunion (France) | 1 year | Hospitals and other private institutions, maternity units, paediatric departments, prenatal screening, prenatal diagnosis centres, pathology laboratories, paediatric surgery and cardiology units | Cytogenetic laboratories, medical genetics | 329.8 [316.1 - 343.8] |
| Wielkopolska (Poland) | <2018: 2 years, ≥2018: 18 years | Neonatology, obstetrics, paediatric surgery, orthopaedics, paediatrics, paediatric cardiology, ophthalmology, paediatric neurology, paediatric otolaryngology, intensive paediatric therapy, primary care, pathomorphology, lung diseases, endocrinology and paediatric diabetes, child and adolescent psychiatry and voivodship branches of the national health fund | Clinical genetics | 260.4 [252 - 269.1] |
| French West Indies (France) | 1 year | Maternity units, paediatric department, prenatal screening, paediatric surgery, paediatric cardiology, neurology, hospital discharge registers | Cytogenetic laboratories, medical genetics | 283.8 [267.6 - 300.6] |
| Valencian Region (Spain) | 1 year | Public and private hospital discharge records, perinatal mortality registry of Comunitat Valenciana, induced abortion registry of Comunitat Valenciana, metabolic disorder registry | Medical genetics, genetic laboratories | 243.9 [236.7 - 251.2 |
| Brittany (France) | 1 year | Prenatal screening, maternity units, neonatologist and paediatric departments, paediatric surgery, echocardiology laboratories, pathology laboratories, paediatric radiology, multidisciplinary committee of prenatal diagnosis (CPDP), Patient Care Classification System Database, as completeness control tool | Medical genetics, cytogenetic laboratories | 392.5 [382.8 - 402.5] |
| Pleven (Bulgaria) | 1 month, for selected anomalies (heart defects, urogenital defects etc.) 1 year | Active screening conducted by neonatologists, obstetricians, clinical geneticists. Hospital records (departments of obstetrics and gynaecology, neonatology, paediatrics), records of pathological meetings, stillbirths register, data from pathological examinations. | Medical genetics/cytogenetic records. The registry has direct access to the cytogenetic laboratory. | 217.9 [190.3 - 248.5] |
| Trento (Italy) | 1 year | Birth attendance certificate (CedAP), hospital discharge records (SDO), the rare diseases registry, the cancer registry, dataset of foetal autopsies of the multizonal anatomy and pathological histology and cytodiagnostics of the APSS of Trento | Database of prenatal and postnatal genetic counselling (medical genetics unit of the APSS of Trento) | 291.7 [268.6 - 316.1] |

DQI, data quality indicator; MCA, multiple congenital anomalies

^a^ Information from registry descriptions on EUROCAT website, see <https://eu-rd-platform.jrc.ec.europa.eu/eurocat/eurocat-members/registries_en>

^b^ Information from EUROCAT Data Quality Indicators (DQI) over birth years 2019-2023 calculated in 2025, see https://eu-rd-platform.jrc.ec.europa.eu/system/files/public/eurocat/20250904_DQI_2019-2023.pdf
